# Development and Implementation of an Ambulatory Orders-Based Ophthalmology Imaging Workflow

**DOI:** 10.1007/s10278-025-01660-x

**Published:** 2025-09-08

**Authors:** Michael K. Oswald, Cieara Presley, Laurie A. Perry, JaTawna Bush, Bessie Ganim, David Hulefeld, Jay Patel, Melissa Scott, Veeral S. Shah, Evan Slavik, Sarah Smith, Kelli Vieson, Fred Walker, Alexander J. Towbin

**Affiliations:** 1 Department of Radiology, University of Cincinnati, Cincinnati, OH USA; 2 Department of Radiology, Cincinnati Children’s Hospital, Cincinnati, OH USA; 3 Information Services, Cincinnati Children’s Hospital, Cincinnati, OH USA; 4 Division of Pediatric Ophthalmology, Cincinnati Children’s Hospital, Cincinnati, OH USA

**Keywords:** Enterprise imaging, DICOM, Ophthalmology, Eye care

## Abstract

**Background:**

Ocular imaging is essential to the diagnosis and management of eye disease, yet standardized imaging workflows remain underdeveloped in the eye care setting. This manuscript describes the design and implementation of an orders-based imaging workflow for ambulatory ophthalmology integrated with the electronic health record and enterprise imaging systems.

**Methods:**

We developed a DICOM-compliant workflow for pediatric ophthalmology imaging that supports HL7 integration, DICOM modality worklists, and enterprise archive storage. Workflow steps were automated where possible, including imaging order placement, metadata application, and documentation. Pre- and post-implementation workflows were evaluated for efficiency, measuring mouse clicks, keystrokes, and time to completion. Imaging study volumes and billing data were collected following implementation.

**Results:**

Thirteen imaging devices across six modalities and five locations were integrated. The new workflow reduced manual data entry and enabled structured documentation. Post-implementation, 16,267 imaging studies were completed, generating $11.4 million in billed charges. Workflow efficiency improved, with keystrokes and mouse clicks reduced by 40–86% depending on modality. Time savings were observed in most modalities, although optical coherence tomography and B-scan ultrasound required more time due to order-specific imaging steps.

**Conclusion:**

An orders-based imaging workflow for ophthalmology can improve efficiency, standardization, and interoperability while enabling proper image archiving and billing. Challenges remain with vendor variability in DICOM support and image output formats. These findings support broader adoption of standardized imaging practices in ophthalmology.

## Introduction

Imaging plays a crucial and expanding role in ophthalmology. In-office ocular imaging is now routinely employed for a variety of purposes, including screening, diagnosis, monitoring, procedural planning, and documentation of numerous eye diseases [1]. Despite its importance, standardization of ocular imaging workflows has been a persistent challenge [2].

In many medical specialties where imaging is integral, the Digital Imaging and Communications in Medicine (DICOM) standard has been adopted. DICOM facilitates vendor-neutral encoding, transmission, and storage of images [3]. Unfortunately, the widespread adoption of the standard has lagged in ophthalmology. As a result, ocular imaging data often lack interoperability, which can lead to inefficient eye care workflows and can negatively impact patient care.

In many instances, imaging data is accessible only through proprietary, vendor-specific viewers, leading to data silos, and ophthalmologists often find themselves viewing images in isolation from the electronic health record (EHR) that contains vital medical history. Moreover, the quality of these images is frequently compromised because native data is commonly compressed to reduce file sizes [4]. The inability to effectively share and access high-quality patient information across EHRs, imaging management systems, and broader medical networks remains a significant challenge in ocular imaging.

Even with the recognized benefits of the DICOM standard and its support from ophthalmology organizations, including the American Academy of Ophthalmology (AAO), the National Eye Institute (NEI), and the Royal College of Ophthalmologists (RCOpth), significant obstacles hinder its full adoption in clinical practice [5–7]. Many vendors are hesitant to fully implement DICOM, opting instead to achieve “DICOM compliance” by generating encapsulated portable document format (PDF) files that merely suggest adherence to DICOM standards without retaining the raw data [8]. Because many eye care facilities are independent from large hospital systems, this reluctance is partly due to the added cost of purchasing and supporting an imaging viewer and archive.

There are also specific workflow challenges unique to ophthalmology. For instance, because ocular imaging modality vendors sell all-in-one solutions, the modalities are often not connected to the electronic health record via Health Level 7 (HL7) interfaces. As a result, imaging technologists must manually enter patient demographic information on devices.

In this paper, we describe our experience implementing an EHR-based imaging workflow in a pediatric ophthalmology clinic. Historically, this clinic was tasked with manually entering patient demographics, printing hard copy images and patient data, and was plagued by numerous inefficient data storage and sharing processes. Our aim was to increase the workflow efficiency of the staff, incorporate DICOM modality worklists to enhance patient safety, improve the storage and retrieval of imaging data, enable ophthalmologist image interpretation workflows, optimize and automate billing protocols, and enable the entire ophthalmology record to be seamlessly integrated with the hospital’s EHR and enterprise imaging archive.

## Materials and Methods

### Systems and Settings

This project occurred in the Division of Pediatric Ophthalmology at a large, quaternary care children’s hospital. The ambulatory ophthalmology clinic cares for over 22,000 infants, children, and adolescents per year. Visual testing, diagnostics, and treatments are available in the diagnosis and management of visual impairments affecting the pediatric population.

Our organization has a large enterprise imaging program that aims to build efficient workflows that enable users to “capture, index, manage, store, distribute, view, exchange, and analyze all clinical imaging and multimedia content [9–11].” We implemented an enterprise imaging archive (Merge; Merative, Ann Arbor, MI) in 2013. The archive serves as a single point of storage for all imaging studies, including those obtained in radiology, cardiology, and other specialties within our institution. An enterprise viewer (Merge Universal Viewer; Merative, Ann Arbor, MI) was launched in 2016. The viewer allows providers to view images obtained by any specialty [12]. Users can access the enterprise viewer via a direct login or a link in the EHR (Epic Systems, Verona, WI).

### Requirements

Our guiding principle was to create a standard process enabling ophthalmology providers to efficiently perform, document, store, and bill for imaging studies across multiple different modalities and procedures. To achieve this, we set requirements for each modality and for the EHR build. First, our enterprise imaging governance team, led by the Associate Chief Medical Information Officer, Chief Technology Officer, and Senior Director of Enterprise Imaging, decided that ocular imaging devices must be able to meet hospital security requirements, create DICOM standard images, use a DICOM modality worklist (MWL), and send DICOM images to the enterprise imaging archive, and be viewed using the enterprise viewer. We believed the DICOM images would enable the routine storage and consumption of obtained images. Using a DICOM MWL would allow providers to apply standard patient demographic metadata to the images efficiently.

The implementation team, comprised of enterprise imaging and EHR analysts and clinical engineers, then worked with clinical providers to identify the requirements of the EHR build. Ideally, imaging workflow should be initiated within standard clinical or procedural workflow appropriate to the context of care. The number of workflow steps was expected to be minimal, ideally decreasing the total number of mouse clicks or keystrokes required to complete the procedure.

## Interventions

### Discovery

During discovery, the enterprise imaging implementation team collaborated with the Ophthalmology division physicians and technologists to gain access to the modalities used for capturing ocular images. This enabled the team to evaluate the devices’ network connectivity, DICOM functionality, and adherence to security protocols. When evaluating the DICOM functionality, the enterprise imaging implementation team looked for and assessed the ability to query for a DICOM MWL and create DICOM images. The implementation team worked with the modality vendors to explore and potentially correct any perceived deficiencies.

Next, the implementation team shadowed the imaging technologists and ophthalmologists to observe and document their current-state workflow. During this step, the team identified that imaging could occur as part of a clinic visit or independent of other clinical activities.

Finally, the team worked with the Ophthalmology division’s business director to compile a list of the procedures and billing codes performed on each device. All standard or structured diagnostic and procedural notes were gathered.

### Assessment

The new EHR-driven workflow was assessed to determine efficiency, use and impact in the ophthalmology clinic. Using a standard patient, an imaging research fellow, an ocular imaging technologist, and an enterprise imaging analyst worked together to count the number of clicks and keystrokes required to perform the workflow from the time of order entry through the time of image acquisition. The time required to complete this work was also recorded. The assessment was performed for both the pre-existing and new workflows. To standardize the assessment, the patient’s name was seven characters in length. Their medical record number was eight characters, and their date of birth was eight characters, including the backslashes.

To assess the impact of the implementation, the number and type of imaging studies were tabulated, and the US dollars (USD) billed were reported from the implementation of the new workflow until December 31, 2024. Because images were not stored before the implementation of the new workflow, the study volumes and USD billed are only reported after the implementation of the new workflow.

## Results

### Workflow

The original clinical imaging workflow was disorganized, complex, and prone to error. Technologists needed to manually enter demographic and clinical data for each patient on each device. After imaging was complete, the technologists printed the images and results on paper for later review by ophthalmologists. Once reviewed and a clinical note was created, the paper copy of the images was scanned for inclusion in the EHR.

The new EHR-based, orders-driven workflow for ophthalmology begins when the patient arrives for their scheduled appointment. If the ophthalmologist determines that imaging is needed, they can select relevant imaging orders from a drop-down menu containing all potential ophthalmology procedures identified during the discovery phase. The selected orders launch forms specific to the imaging modality. Because imaging orders can be selected from the drop-down menu in advance of the clinic visit through standard ambulatory pre-visit workflow, the orders are not released until the patient is registered. Once registered, the patient’s status changes to “Arrived,” and the patient appears on the technologist’s worklist. The technologist releases the order(s) to the modality, selects the patient from the MWL, and captures imaging for the patient. This order of operation ensures that all relevant patient and study metadata is automatically applied to the images. Once imaging is complete, the images are automatically transmitted to the picture archiving and communication system (PACS) and auto-forwarded to the enterprise imaging archive. After completing the imaging study, the technologist then returns to the patient’s EHR to finalize the study documentation.

This EHR-driven workflow also allows the ophthalmologist to write notes associated with the imaging study. If a standardized note exists for the study, it is automatically applied to the procedure. The ophthalmologist can then add any unique and relevant information based on the study’s findings. Once the note is signed, a link is generated within the imaging result in the EHR that enables the launch of the enterprise viewing program. Non-ophthalmology users can view the results on both the imaging tab and the results tab of the patient’s chart.

### Implementation

Thirteen devices spanning six different imaging modalities at five distinct clinic locations were configured to use the new EHR-driven workflow (Table 1). A total of nine unique orders for imaging procedures were created and used during the first phase of implementation. The integrated modalities included B-scan ultrasound, Optos fundus, corneal topography, optical coherence tomography, slit lamp, and visual fields. Each modality had a unique workflow and imaging output. Specifically, three modalities (B-scan ultrasound, Optos fundus, and slit lamp) were able to output raw images, one (corneal topography) was able to output DICOM secondary capture images, and one (visual field) was able to output DICOM-compliant PDF reports. The optical coherence tomography modality was able to output both raw images and a DICOM-compliant PDF report. The type of output from this modality depended on the imaging procedure.

**Table 1:** Model, vendor information, and the number of each modality integrated into the new ophthalmology workflow

Modality	Model	Vendor	Number of modalities integrated
B-scan ultrasound	**4Sight**	**Accutome Inc**	**9**
Optos fundus	**P200TxE**	**Optos**	**2**
Visual field	**Octopus 900**	**Haag-Streit AG**	**2**
Slit lamp	**BX 900**	**Haag-Streit AG**	**1**
Optical coherence Tomography	**SPECTRALIS**	**Heidelberg Engineering**	**2**
Corneal topography	**Pentacam**	**Oculus**	**1**

During the implementation process, multiple challenges were identified (Table 2). These challenges could be placed into one of three categories: modality challenges, workflow challenges, and imaging output challenges. Modality challenges were the most difficult to address. Many of the challenges stemmed from the vendor’s inexperience with DICOM-based tools like MWL or HL7 interfaces. In one instance, the historical patient’s name data had been using the FIRST NAME LAST NAME format. This differed from the standard LAST NAME, FIRST NAME format. Because of the differences, patient data appeared incorrect and had to be manually reconciled on the machine. For the most part, the modality challenges were addressed in partnership with the vendors.

**Table 2:** Summary of issues addressed during implementation of ophthalmology imaging workflow

Category of issue	Issue	Specific challenge	Resolution	Responsible team(s)
Modality	Inconsistent name formatting	Devices used FIRST LAST format instead of standard LAST, FIRST	Manual reconciliation and updated configuration with vendor	Enterprise imaging, Vendor
Modality	Lack of DICOM modality worklist support	Devices did not support DICOM modality worklist functionality out of the box	Vendor collaboration and configuration updates	Enterprise imaging, Vendor
Modality	HL7 integration limitations	Vendors were inexperienced with HL7 interfaces	Ongoing collaboration and vendor education	Enterprise imaging, Vendor
Workflow	Imaging may occur independently or as part of a clinic visit	Imaging-only appointments could not be scheduled in the same manner as those associated with a clinic visit	Created a testing room so that appointments and orders could be scheduled	Electronic health record
Workflow	New process unfamiliar to users	Technologists unfamiliar with new EHR-driven workflow	Onsite training and continued user support	Enterprise imaging, clinical informatics
Workflow	Longer imaging time for OCT and B-scan	Separate orders increased workflow steps for multiple procedures	Addition of combined imaging orders to streamline process	Enterprise imaging, Ophthalmology
Image output	PDF reports instead of raw DICOM	Several modalities produced DICOM-compliant PDF reports only	Acceptance of PDF output with awareness of limitations	Enterprise imaging
Image output	PACS viewer places PDF reports in report viewer rather than treating it as imaging output	PDF reports file to report viewer, limiting comparison over time	Acknowledgement of limitations; long-term goal to shift to raw image support	Enterprise imaging, Viewer governance

Workflow difficulties were related to the newly created process. These were solved by in-person user training and onsite technical support. Since implementation, the technologists and ophthalmologists have become comfortable with the designed workflow. One of the more complex challenges was developing a process for imaging-only clinic appointments. In the historical workflow, imaging was performed based on a paper order document after the patient was scheduled. In the new workflow, patients are scheduled directly to a testing room, and imaging orders are placed in advance of the visit. The imaging-only workflow then functions similarly to workflows used during standard clinic visits.

Image output difficulties were related to the proprietary format of native imaging files. Many modalities output a DICOM-compliant PDF report rather than raw images. These reports are filed to the report viewer in our PACS and enterprise viewers. While ophthalmologists have become used to the report placement, this location has limited the ability for ophthalmologists to compare imaging over time and has precluded the imaging informatics team’s ability to build robust display protocols within the viewer.

### Assessment

A comparison of the historical and newly implemented processes (Table 3) showed that the new process reduced the number of mouse clicks and keystrokes required to perform and document specific modalities from start to finish by between − 39.7 and − 86.2% depending on the modality. The time (in seconds) needed to complete each study varied. The optical coherence tomography and B-scan ultrasound modalities took longer (+ 65.2 and + 22.5%, respectively) to complete. The time required to perform imaging was shorter on the other modalities, with time reductions ranging from − 64.9 to − 89.4%.

**Table 3:** Comparison between the newly implemented workflow and the old workflow across six common ocular imaging modalities. Clicks represent the total number of mouse clicks and keystrokes to perform and document the specific modalities. Time is measured in seconds

	Pre-existing workflow		New workflow		Percent change with new workflow	
Modality	Number of mouse clicks and keystrokes	Time (s) to complete workflow	Number of mouse clicks and keystrokes	Time (s) to complete workflow	Number of mouse clicks and keystrokes	Time (s) to complete workflow
Corneal topography	29	37	4	12	− 86.2%	− 67.6%
Visual field	68	94	10	33	− 85.3%	− 64.9%
Slit lamp	68	94	11	10	− 83.8%	− 89.4%
Optos fundus	32	23	7	14	− 78.1%	− 39.1%
Optical coherence tomography	58	46	35	76	− 39.7%	65.2%
B-scan ultrasound	33	40	6	49	− 81.8%	22.5%

A total of 16,267 imaging studies were performed between November 15, 2022, and December 31, 2024. The total dollars billed during this time were $11,406,928. Procedure volume data are included in Table 4.

**Table 4:** Ophthalmology imaging modalities, image types, procedures, and volumes for implemented solution between November 15, 2022, and December 31, 2024

Modality	Image type	Procedure	Procedure volume	$ US billed per procedure	Total $ US Billed	Modality volume
**B-scan ultrasound**	***DICOM***	*B-scan ultrasound*	567	$941	$533,547	567
**Optos fundus**	***DICOM***	*Optos fundus photos*	5115	$461	$2,358,015	5283
	***DICOM***	*Fluorescein angiography*	168	$918	$154,224	
**Visual field**	***PDF-report(s)***	*Visual field, extended*	1716	$1125	$1,930,500	1716
**Slit lamp**	***DICOM***	*Slit lamp photography*	230	$250	$57,500	230
**Optical coherence tomography**	***PDF-report(s)***	*OCT, Optic nerve*	4453	$762	$3,393,186	8241
	***PDF-report(s)***	*OCT, Retina*	3766	$773	$2,911,118	
	***DICOM***	*OCT, Anterior segment*	22	$254	$5588	
**Corneal topography**	***PDF-report(s)***	*Corneal topography*	230	$275	$63,250	230
**Sum totals**			**16,267**		**$11,406,928**	**16,267**

## Discussion

Recently, the AAO and NEI have acknowledged the need for a standardized approach to ocular imaging [6, 13]. The AAO has advised device manufacturers to provide machine-readable, discrete data for user-selected reports and to use lossless compression to preserve raw data for a wide range of devices [14]. Despite these recommendations, non-compliant devices remain.

Here, we describe our EHR-based imaging workflow and provide an analysis detailing its impact on the practice. Our workflow is designed to be as efficient as possible, leveraging automation to limit the number of potential keystrokes and mouse clicks by an end user. Not only does this favor efficiency, but it also promotes accuracy and workplace well-being [15–17]. Specific workflow pressure points that can be alleviated through automation include ad-hoc order creation from a pre-determined list of orders, automated scheduling of procedures, automated begin- and end-examination steps, DICOM MWL to populate patient demographics, and the use of pre-populated auto-text options for imaging results. These changes promote not only efficiency but also better data consistency and the prevention of manual entry errors.

During workflow design, we had to determine the best method to drive the imaging workflow: orders or encounters [18]. After some discussion, we decided on an orders-based workflow. We made this decision for several reasons. First, we believed that an orders-based workflow would give us the most flexibility. Ophthalmology practices perform many different imaging studies on multiple modalities. We thought that an orders-based workflow would best drive automated billing and unique results reporting. Additionally, we were concerned that an encounters-based workflow would not be scalable given the potential combination of various imaging studies, the differences in potential clinic visit types, and the different settings available for imaging to be performed. We attempted to mitigate the burdens of orders by automating as many steps as possible. For example, the order entry process is automated by selecting the procedure from a drop-down worklist; the imaging order automatically progresses through begin-exam and end-exam steps once the order is released; and results creation is semi-automated using standard report content. We believe our final workflow effectively balances workflow efficiency and clinical specificity.

We identified multiple challenges while implementing this workflow. These challenges mostly centered around the modality vendor’s inexperience with DICOM-based tools and the modality’s image output. We believe that other sites will have similar challenges as they begin to implement eye-imaging workflows. These challenges will continue until the modality vendors commit to consistently outputting DICOM images.

While the new workflow was generally more efficient, it resulted in longer imaging times for both optical coherence tomography (OCT) and B-scan ultrasound. This increase occurred despite a reduction in mouse clicks and keystrokes. The delay in OCT imaging stemmed from the need to access each exam individually. Previously, technologists could combine all required imaging under one encounter. With the new setup of separate imaging orders, technologists had to select the first order from the MWL, complete and send the imaging, then return to the MWL to repeat the process for each subsequent order. This made the workflow more tedious and, at times, hindered imaging, especially in uncooperative pediatric patients. To address this, a combined imaging order was added in a later phase of the project to streamline the process and improve success.

There are several limitations to our workflow. First, while we see the overall simplicity of the workflow as a strength, it still requires training. Considering the rare prevalence of such a workflow in the ophthalmology community, new providers will require training. Second, while we were able to implement the workflow for the ambulatory setting, it is not yet rolled out for the inpatient and perioperative settings. These orders have been built in the EHR but will require device configuration and testing prior to implementing the second phase of the desired workflow.

Finally, while we attempted to demonstrate the impact of the new workflow on employee work and dollars billed, these measures are crude surrogates. Because we did not compare the number of studies performed on the billing pre- and post-implementation, we are not able to show if this workflow improved our organization’s ability to bill for services rendered. Additionally, we are not able to determine if the workflow saves time for the ophthalmologists. Anecdotally, our ophthalmologists are happy with the workflow and have asked that it be expanded to other care settings.

In conclusion, we have implemented an efficient orders-based ambulatory workflow for ophthalmology imaging. This workflow has enabled providers to perform multiple ocular imaging studies, document their results, store the images, and bill for the services. Although this process is specifically designed for the EHR, we believe the principles are generalizable and can be applied across many different systems.
